# Selecting for BRCA1 testing using a combination of homogeneous selection criteria and immunohistochemical characteristics of breast cancers

**DOI:** 10.1186/1471-2407-9-360

**Published:** 2009-10-10

**Authors:** GianMaria Miolo, Vincenzo Canzonieri, Clelia De Giacomi, Lara Della Puppa, Riccardo Dolcetti, Davide Lombardi, Tiziana Perin, Simona Scalone, Andrea Veronesi, Alessandra Viel

**Affiliations:** 1 Department of Molecular Oncology and Translational Research, Division of Experimental Oncology 1, Centro di Riferimento Oncologico, National Cancer Institute, Aviano, Italy; 2 Department of Senology, Division of Medical Oncology C, Centro di Riferimento Oncologico, National Cancer Institute, Aviano, Italy; 3 Department of Diagnostic Laboratories and Cell Therapy, Division of Pathology, Centro di Riferimento Oncologico, National Cancer Institute, Aviano, Italy; 4 Department of Medical Oncology, Cancer Biommunotherapy Unit, Centro di Riferimento Oncologico, National Cancer Institute, Aviano, Italy

## Abstract

**Background:**

BRCA1 gene-related tumours are more frequently estrogen receptor (ER) and progesterone receptor (PR) negative with a lower prevalence of human epidermal growth factor receptor 2 (HER2) overexpression or amplification. We evaluated the effectiveness of a combination of homogeneously selected criteria and immunohistochemical (IHC) characteristics of Familial Breast Cancers (FBCs) in detecting BRCA1 mutation carriers.

**Methods:**

Primary breast tumours from 93 FBC patients defined by specific eligibility criteria, based on personal and familial tumour history, were evaluated by Allred's method. The BRCA1 molecular analysis, including Multiplex Ligation-dependent Probe Amplification (MLPA), was considered as the gold standard assay.

**Results:**

A total of 10 BRCA1 pathogenetic mutations was found. With the exclusion of the tumours characterized by double positive receptorial status and/or strong HER2 positivity (3+), we identified 22 patients, 10 of whom resulted as BRCA1 mutation carriers. The sensitivity, specificity, positive and negative predictive values were 100%, 83.3%, 45.4% and 100% respectively.

**Conclusion:**

Our findings suggest that the IHC analysis by Allred's method improves our ability to select patients for BRCA1 testing.

## Background

One of the still unexplained features of BRCA1-mutant breast tumours is their frequent lack of ER expression; in fact, up to 90% of these tumours exhibit a loss of ER expression [1,2]. In addition, BRCA1-associated breast cancers frequently show a low prevalence of HER2 overexpression or amplification [3-5].

Patients with a strong family history of breast and/or ovarian cancer are counselled to receive genetic testing for mutations in the BRCA1/BRCA2 genes. The selection of patients is crucial and relies mostly on family history and phenotype ascertainment [6-8]. In general, only 20-40% of screened families, even using rigid selection criteria, have positive mutation results [9,10]. In particular, the rate of mutations in Italian breast and/or ovarian cancer families is rather controversial and ranges from 8% to 37% [11]. Moreover, it has been recently pointed out that only less than 10% of cases with a positive family history harbour a BRCA1 mutation [12].

In this study, we evaluated the effectiveness of a combination of selected eligibility criteria and IHC characteristics of breast cancer to identify patients who have a higher probability of being BRCA1 mutation carriers.

## Methods

From September 2002 to October 2008, a total of 93 female index cases affected with breast cancer complying with the eligibility criteria for BRCA testing in use at the Centro di Riferimento Oncologico, National Cancer Institute, Aviano, Italy were enrolled in the study [13]. Cases affected with ovarian cancer and male breast cancer were excluded. Written informed consent for genetic testing was obtained from all the participants. The study fully conformed to the Declaration of Helsinki and to the current Italian Legislation and was also approved by the Ethical Committee of the Institute.

Genomic DNA was purified from blood samples obtained from each proband. Screening for mutations in the BRCA1/BRCA2 genes was carried out by Denaturing High Performance Liquid Cromatography and direct sequencing techniques. Primer sequences and PCR conditions have been previously described [14-16]. Germline rearrangements of BRCA1/BRCA2 genes were also analysed by Multiple Ligation-dependent Probe Amplification (MLPA). Missense, silent, and intronic mutations, whose clinical significance have not yet been reported, were classified as genetic variants of uncertain significance (unclassified variant, uv) in accordance with the BIC database [17].

Ventana Medical Systems clone 6F11 (Ventana Medical Systems, Inc., Tucson, AZ) for the evaluation of the ER and Ventana Medical Systems clone 1E2 for the evaluation of the PR were used. With the Allred score, the proportion score (PS) and the intensity score (IS) are assessed in six and four grades, respectively 0-5 and 0-3, then the total score is assessed in eight grades (0 and 2-8) [18]. Tumours with an IHC total score of 3 or more were reported as positive. The IHC analysis of HER2 was performed by staining with the rabbit anti-human HER2 antibody clone 4B5 (Ventana Medical Systems). A score index of 0, 1, 2 and 3 was used, corresponding respectively to negative, weak, moderate and strong staining intensity, and the percentage of positive cells at each intensity was estimated subjectively.

The sensitivity, specificity, and positive and negative predictive values were calculated from the results of the IHC analysis to predict for BRCA1 mutations.

## Results

In total, 19 different BRCA1/BRCA2 deleterious mutations were identified in the 93 unrelated patients. Ten (10.8%) patients resulted BRCA1 mutation carriers whereas in nine (9.7%) a BRCA2 mutation was found (Tables 1 and 2). The prevalence of BRCA1 large genomic rearrangements accounted for 20% (2/10) of the BRCA1 gene mutation spectrum. No large deletions or duplications involving the BRCA2 gene were observed. In this series, all BRCA1 tumours showed a double or a single negative hormonal receptorial status, whereas HER2 was invariably negative or only weakly expressed (Table 1).

**Table 1:** BRCA1 group and IHC tumour characteristics.

ID pts	Age at diagnosis	DNA	Mutation effect	Grade	° ER (score)	° PR (score)	§HER2	^ BRCAPRO BRCA1	^ BRCAPRO BRCA2	^ BRCAPRO total	*BC and CBC cases	*OC cases
BR332	42	633delC	233X	3	- (2)	- (0)	-	0.975	0.013	0.987	1 (27)	2 (35, 56)

BR334	26	2428C>A	S770X	3	- (0)	- (0)	-	0.658	0.091	0.748	1 (53)	1 (75)

BR341	45	del 9-19	del ex. 9-19 ter 184	3	- (0)	- (0)	-	0.320	0.524	0.841	3 (37, 48 M, 52)	/

BR352	37	del 23-24	unpredictable	3	- (0)	- (0)	1+	0.777	0.075	0.850	1 (29+42 CBC)	/

BR384	50+56 CBC	795delT	233X	3	- (0)	- (0)	1+/2+	0.529	0.085	0.613	2 (56, **57**)	1 (57)

BR386	40	1806C>T	Q563X	3	- (0)	- (0)	1+/2+	0.311	0.048	0.358	/	1 (69)

BR392	31	795delT	233X	3	- (0)	- (0)	-	0.813	0.066	0.876	1 (46+48 CBC)	/

BR441	46	5181delGTT	1688delV	3	- (0)	- (0)	-	0.166	0.036	0.202	3 (**46**, 47, 48)	1 (63)

BR507	22	633delC	233X	3	- (0)	- (0)	1+	0.091	0.015	0.106	/	/

BR587	38+60 CBC	300T>G	C61G	3	- (0)	- (0)	1+	0.922	0.063	0.984	2 (**40, 43**)	1 (60)

BC = breast cancer; CBC = controlateral breast cancer; OC = ovarian cancer; / = no cases; M = male. The ages at diagnosis of the first cousins are in boldface type; ° IHC Allred total scores of 3 or more were reported as positive. §HER2 IHC scores: - negative, 1+ weak, 2+ moderate, 3+ strong. ^ Probability of mutation calculated by BRCAPRO software http://www.isds.duke.edu/~gp/brcapro.html; * number of additional tumours in the family and ages of onset in parentheses.

**Table 2:** BRCA2 group and IHC tumour characteristics.

ID pts	Age at diagnosis	DNA	Mutation effect	Grade	° ER (score)	° PR (score)	§HER2	^ BRCAPRO BRCA1	^ BRCAPRO BRCA2	^ BRCAPRO total	*BC, CBC and BBC cases	*OC cases
BR288	36	9326insA	3043X	3	+ (6)	+ (5)	1+	0.003	0.026	0.029	/	1 (44)

BR295	54	657insC	157X	2	+ (8)	+ (5)	-	0.029	0.405	0.434	2 (41+43 CBC, 48)	/

BR312	47	IVS16-2A>G	Exon skipping	3	+ (5)	+ (3)	1+	0.002	0.365	0.367	2 (47, 77 M)	/

BR342	44+52 CBC	9106C>T	Q2960X	3	+ (8)	+ (3)	2+	0.006	0.182	0.187	2 (59, 68+75 CBC)	/

BR360	43	IVS23+1G>T	Exon skipping	3	+ (4)	+ (6)	2+/3+	0.088	0.553	0.640	1 (30 BBC)	/

BR382	44+63 CBC	7507del7	2168X	3	+ (6)	+ (8)	1+	0.002	0.061	0.063	1 (35)	/

BR410	24	9325delA	3061X	2	+ (7)	+ (4)	1+	0.170	0.557	0.727	1 (47)	2 (54, 73)

BR450	48	4450C>T	Q1408X	1	+ (7)	+ (7)	-	0.010	0.044	0.054	2 (52, 55)	2 (38, 55)

BR571	31	3036del4	959X	2	+ (3)	+ (6)	3+	0.001	0.037	0.038	3 (50, 50, 61)	/

BC = breast cancer; CBC = controlateral breast cancer; BBC = bilateral breast cancer; OC = ovarian cancer; / = no cases; M = male. ° IHC Allred total scores of 3 or more were reported as positive. §HER2 IHC scores: - negative, 1+ weak, 2+ moderate, 3+ strong. ^ Probability of mutation calculated by BRCAPRO Software http://www.isds.duke.edu/~gp/brcapro.html; * number of additional tumours in the family and ages of onset in parentheses.

Altogether, 21 cases showed an uv alteration of BRCA1 and/or BRCA2 genes: 7 BRCA1 uv, 7 BRCA2 uv, 5 cases with a variant in both genes, 1 triple BRCA1 uv and 1 double BRCA2 uv associated with a BRCA1 uv, respectively (Table 3).

**Table 3:** BRCA1 and/or BRCA2 uv group and IHC tumour characteristics.

	ID pts	Age at diagnosis	DNA	Mutation effect	Grade	° ER (score)	° PR (score)	§HER2	^ BRCAPRO BRCA1	^ BRCAPRO BRCA2	^ BRCAPRO total	BC, CBC and BBC cases	OC cases
BRCA1 uv													

	BR417	30	1186A>G	Q356R	2	+ (3)	- (0)	3+	0.015	0.040	0.055	1 (46)	/

	BR427	48	4274A>G	L1385L	2	+ (7)	+ (7)	1+	0.000	0.010	0.011	1 (44)	/

	BR437	50	2351T>G	A744A	1	+ (5)	+ (3)	1+	0.000	0.014	0.015	3 (50, **55**, 59)	/

	BR523	48 BBC	IVS20+60ins12		2	+ (6)	+ (7)	1+	0.018	0.245	0.263	3 (50,50, **52**)	/

	BR570	47	5221T>C	L1701P	3	- (0)	+ (3)	3+	0.094	0.169	0.264	2 (49+52 CBC, 56)	/

	BR647	49	1186A>G	Q356R	3	+ (8)	+ (7)	1+	0.005	0.010	0.015	3 (**65**, 67, **70**)	/

	BR658	43	1186A>G	Q356R	3	+ (6)	+ (6)	2+	0.001	0.069	0.070	3 (50+63 CBC, 57, 60*)	/

	BR665	65 BBC	655A>G	Y179C	2	+ (5)	+ (5)	-	0.001	0.004	0.004	3 (60, 80, 80)	/

			1575T>C	F486L									

			1767A>G	N550H									

BRCA2 uv													

	BR376	55	IVS2-7T>A		2	+ (6)	+ (3)	1+	0.000	0.008	0.008	2 (45, 90)	/

	BR390	51 OC+54 BBC	6328C>T	R2034C	2	+ (5)	+ (8)	-	0.130	0.648	0.777	3 (34, 39, **40**)	/

	BR458	44+49 CBC	5972T>C	T1952I	2	+ (7)	+ (5)	-	0.025	0.433	0.459	2 (50 BBC, **70**)	/

	BR486	56	2457T>C/ 9329A>G	H743H/R3034Q	3	+ (5)	+ (8)	-	0.000	0.000	0.000	4 (**57, 71**, 75, 90)	/

	BR505	67	6751C>G	Q2175E	1	+ (7)	+ (7)	-	0.018	0.426	0.444	4 (40 BBC, 50 BBC, 53 BBC, 58)	/

	BR538	62	4236G>A	L1356L	2	+ (7)	+ (7)	-	0.001	0.025	0.026	2 (**50 **BBC, 56)	/

	BR587	38+60 CBC	IVS24-113T>G		3	- (0)	- (0)	1+	0.922	0.063	0.984	2 (**40, 43**)	1 (60)

BRCA1/2 uv													

	BR514	57	3238G>A	S1040N	1	+ (7)	+ (8)	-	0.001	0.061	0.062	4 (42, **45**, 49, 55)	/

			IVS4+67A>C 1613A>G	E462G									

	BR552	70	4158A>G	R1347G	3	+ (5)	+ (3)	3+	0.000	0.012	0.012	3 (40, 47, 65)	/

			6328C>T	R2034C									

	BR567	50	IVS4+67A>C		3	+ (6)	+ (7)	-	0.009	0.215	0.225	3 (**37 **BBC, 50 BBC, 50)	1 (**59**)

			2457T>C	H743H									

	BR571	31	IVS9-34T>C		2	+ (3)	+ (6)	3+	0.001	0.037	0.038	3 (**50**, 50, 61)	/

			5972C>T	T1951M									

	BR580	43	3238G>A	S1040N	3	+ (7)	+ (5)	1+	0.001	0.030	0.031	2 (46, 68)	/

			1409A>G	E394A									

	BR640	30	855T>G	L246V	3	+ (7)	- (0)	1+	0.117	0.261	0.378	2 (30, 40)	/

			4296G>A	L1356L									

BC = breast cancer; CBC = controlateral breast cancer; BBC = bilateral breast cancer; OC = ovarian cancer; / = no cases; M = male. The ages at diagnosis of the first cousins are in boldface type; ° IHC Allred total scores of 3 or more were reported as positive. §HER2 IHC scores: - negative, 1+ weak, 2+ moderate, 3+ strong. ^ Probability of mutation calculated by BRCAPRO software http://www.isds.duke.edu/~gp/brcapro.html; * number of additional tumours in the family and ages of onset in parentheses.

To evaluate the sensitivity and specificity of the IHC screening in a selected set of patients, the combination between BRCA1 point mutation screening and MLPA was considered as the gold standard assay. According to the receptorial status, patients were divided into two groups. The first group included all the ER negative and/or PR negative tumours, whereas the second group consisted of the double positive ones. The BRCA1/2 negative mutation group is shown in Table 4.

**Table 4:** non BRCA1/2 mutation group and IHC tumour characteristics.

ID pts	Age at diagnosis	Grade	° ER (score)	° PR (score)	§HER2	^ BRCAPRO BRCA1	^ BRCAPRO BRCA2	^ BRCAPRO total	*BC, CBC and BBC cases	*OC cases
BR271	40	G2	+ (7)	+ (5)	1+/2+	0.002	0.036	0.038	1 (37)	/

BR275	28 BBC	G3	+ (7)	+ (7)	-	0.067	0.578	0.645	1 (55)	/

BR281	42	G3	+ (6)	+ (4)	3+	0.008	0.222	0.230	6 (27, 36, 50, 60, 50 BBC, 60)	/

BR297	54	G3	- (0)	- (0)	-	0.064	0.018	0.081	2 (42, 69)	/

BR304	41	G3	+ (7)	+ (6)	-	0.002	0.058	0.060	2 (52, 64)	/

BR315	46 BBC	G2	+ (7)	+ (7)	1+	0.012	0.160	0.172	1 (49)	/

BR335	64	G2	+ (8)	- (0)	1+	0.002	0.017	0.020	2 (57, 70)	1 (69)

BR338	36	G3	- (0)	- (0)	3+	0.131	0.029	0.160	2 (45, 71)	/

BR339	74	G2	+ (7)	+ (7)	-	0.003	0.085	0.088	3 (39, 61+63 CBC, 66)	2 (54, 64)

BR340	35	G3	+ (7)	- (0)	3+	0.009	0.026	0.035	2 (**43**, 59)	/

BR348	55	G3	+ (7)	- (0)	1+	0.009	0.062	0.071	2 (56, 56)	1 (68)

BR350	42	G3	+ (7)	+ (7)	2+	0.001	0.014	0.015	4 (49, 57, 61*, 70*)	/

BR357	35	G3	+ (6)	- (0)	-	0.002	0.014	0.016	2 (55, 67)	/

BR377	46	G3	+ (7)	+ (6)	-	0.000	0.007	0.007	2 (46, 70)	/

BR380	30	G2	- (0)	- (0)	1+/2+	0.303	0.067	0.370	3 (42, 64, 64)	/

BR381	45	G3	+ (5)	+ (6)	-	0.000	0.004	0.004	2 (45, 85)	/

BR398	50	G2	+ (5)	+ (3)	-	0.000	0.020	0.020	5 (60, 61, 65, 70, 98)	/

BR403	53+57 CBC	G3	+ (7)	+ (7)	-	0.000	0.006	0.007	1 (82+84 CBC)	/

BR408	53	G2	+ (6)	+ (4)	-	0.000	0.002	0.002	3 (71*, 85*, 67°)	/

BR414	60	G2	- (2)	+ (4)	-	0.003	0.101	0.104	2 (73M, 50)	/

BR418	27	G3	- (0)	- (0)	3+	0.093	0.015	0.108	/	/

BR422	55	G3	+ (6)	- (2)	-	0.002	0.014	0.016	2 (50, 53)	/

BR424	45	G2	+ (5)	+ (7)	2+	0.006	0.113	0.119	1 (49+59 CBC)	/

BR425	27	G2	+ (3)	+ (5)	3+	0.001	0.012	0.013	/	/

BR439	52	G3	- (0)	- (2)	-	0.052	0.030	0.082	3 (**36**, 60, 70)	1 (78)

BR445	54	G1	+ (6)	+ (3)	1+	0.004	0.067	0.072	2 (**45**, 48+52 CBC)	/

BR446	50	G2	+ (7)	+ (4)	1+	0.000	0.008	0.008	1 (40)	/

BR447	65	G2	+ (8)	+ (6)	-	0.001	0.045	0.046	2 (58, 79)	/

BR449	50	G2	+ (6)	+ (7)	-	0.001	0.020	0.021	1 (37)	/

BR457	53	G3	+ (4)	- (2)	3+	0.000	0.003	0.004	2 (55, 55)	/

BR463	48	G3	+ (6)	+ (5)	-	0.001	0.032	0.033	1 (67 BBC)	/

BR480	53	G2	+ (7)	+ (7)	-	0.004	0.135	0.139	3 (31, 50, 53)	/

BR484	57	G2	+ (7)	+ (7)	1+	0.000	0.014	0.014	2 (55, 73+78 CBC)	/

BR494	58	G3	+ (5)	- (0)	-	0.020	0.352	0.372	4 (40, 43, 45, NA M)	/

BR498	46	G2	+ (5)	+ (7)	-	0.005	0.837	0.841	4 (42 BBC, 48, 49M, 74)	/

BR506	55	G1	- (0)	+ (5)	-	0.000	0.012	0.012	3 (62, 30, 76)	/

BR535	56	G3	+ (7)	+ (7)	1+	0.000	0.019	0.019	3 (47, 53, NA)	/

BR542	45	G3	+ (7)	+ (7)	1+	0.007	0.112	0.119	2 (36,62)	/

BR545	63	G2	+ (7)	+ (6)	1+	0.000	0.003	0.003	2 (64, 70)	/

BR549	36	G3	+ (6)	+ (5)	1+	0.013	0.142	0.155	1 (36)	/

BR553	54	G2	+ (7)	+ (6)	1+	0.011	0.486	0.497	2 (49, 65)	1 (55)

BR558	45	G1	+(7)	+ (8)	1+	0.000	0.029	0.030	3 (48, 78, 78)	/

BR560	78	G2	+ (7)	+ (3)	1+	0.000	0.025	0.025	3 (45, 48, 78)	/

BR569	41	G2	+ (7)	+ (8)	-	0.114	0.072	0.185	/	2 (60, 60)

BR582	27	G3	+ (6)	+ (8)	-	0.001	0.016	0.016	/	/

BR584	42	/	+ (5)	+ (6)	1+	0.001	0.017	0.018	3 (40, 54, 69)	**/**

BR592	67	G2	+(7)	+(7)	2+	0.001	0.035	0.036	4 (48, 52, 55, 60)	**/**

BR593	48	G3	+(7)	+ (3)	1+	0.088	0.242	0.330	3 (33 BBC, 65, 70)	1 (24)

BR599	39+44 CBC	G3	+ (6)	- (0)	3+	0.187	0.370	0.557	2 (44, 60)	**/**

BR603	40	G2	+ (7)	+ (7)	-	0.001	0.032	0.033	1 (36)	**/**

BR615	49	G1	+ (7)	+ (7)	1+	0.000	0.004	0.004	2 (49, 50)	**/**

BR649	80	G2	+ (7)	+ (6)	1+	0.001	0.404	0.406	2 (31, 44 BBC)	**/**

BR650	47	G3	+ (6)	- (0)	-	0.004	0.017	0.021	1 (45)	**/**

BR656	43	G2	+ (6)	+ (7)	1+	0.003	0.269	0.272	3 (40, 44, 52)	**/**

BR666	56	G2	+ (8)	+ (6)	1+	0.132	0.201	0.332	3 (39, 50, 51)	3 (49, 54, 61)

BC = breast cancer; CBC = controlateral breast cancer; OC = ovarian cancer; / = no cases; M = male. The ages at diagnosis of the first cousins are in boldface type; ° IHC Allred total scores of 3 or more were reported as positive. §HER2 IHC scores: - negative, 1+ weak, 2+ moderate, 3+ strong. ^ Probability of mutation calculated by BRCAPRO software http://www.isds.duke.edu/~gp/brcapro.html; * number of additional tumours in the family and ages of onset in parentheses.

Twenty-nine tumours did not show a positive staining for ER and/or PR hormone receptors. Notably, 10 of these negative cases were from BRCA1 mutation carriers (Table 5). Furthermore, not considering the cases showing a strong HER2 positivity (3+), the mutation screening could have been performed in only 22 patients, detecting the same number of BRCA1 mutations (Table 6).

**Table 5:** Relation between breast cancer receptorial status and BRCA1 mutant genotype.

Receptorial status	BRCA1 carriers	non-BRCA1 cases	Total
° ER- and/or PR-	10	19	29

ER+/PR+	-	64	64

Total	10	83	93

Sensitivity 100%; Specificity 77.1%; Predictive Positive Value 34.4%; Predictive Negative Value 100%; ° IHC Allred total scores of 3 or more were reported as positive.

**Table 6:** Relation between breast cancer receptorial status and BRCA1 mutant genotype excluding HER2 3+ cases.

Receptorial status	BRCA1 carriers	non-BRCA1 cases	Total
° ER- and/or PR-	10	12	22

ER+/PR+	-	60	60

Total	10	72	82

Sensitivity 100%; Specificity 83.3%; Predictive Positive Value 45.4%; Predictive Negative Value 100%; ° IHC Allred total scores of 3 or more were reported as positive.

The detection rate of BRCA1 mutations was shown to increase from 10.8% (10/93) to 45.5% (10/22), thus allowing the minimization of the number of patients who should undergo BRCA1 mutation screening.

## Discussion

BRCA1-associated tumours have a high probability of being ER negative (up to 90%), PR negative (79%) and with a low frequency of HER2 expression [4,5,19-21]. Physical co-deletion of BRCA1 and HER2 loci on chromosome 17q, occurring as a second somatic inactivating hit, may be at least partly responsible for the low incidence of HER2 expression oramplification in BRCA1-associated carcinomas [22-24].

In the present series, four of the five triple negative tumours (80%) harboured BRCA1 mutations. Consistently with these data, the screening carried out by Musolino et al detected 10 BRCA1 mutations: seven of the eight IHC evaluated BRCA1-related tumours had a triple negative phenotype (87%) and all were negative for HER2 expression [25].

We report an overall prevalence of BRCA1 mutations of 10.8% (10/93). The FBC screening procedure based on the selection of women who were ER and/or PR negative and with weakly expressed HER2, showed a sensitivity in BRCA1 mutation carriers of 100% and a specificity of 83.3%, with a positive predictive value of 45.4% and a negative predictive value of 100%. Moreover, all BRCA1 mutation carriers were identified by using IHC screening, thus confirming that the Allred's method can be used to accurately predict the probability of being a carrier of the BRCA1 mutation.

The IHC test has a very high sensitivity and specificity, and may substantially improve the detection of BRCA1 pathogenetic mutations in families with hereditary breast cancer.

It has been suggested that the IHC analysis of ER may be a new powerful predictor of the BRCA1 mutation status. Estimations show that the probability of being a BRCA1-mutation carrier in a woman with FBC diagnosed before the age of 35 years with a grade 3 tumour and ER negative status is 25%. However, the probability falls to only 5% if the tumour is ER positive [1,5].

The immunophenotypic features of breast carcinomas arising in BRCA1 mutation carriers have also been evaluated by Palacios et al, showing that only 25% of BRCA1-associated tumors were ER positive and PR negative, whereas all cases were HER2 negative [24].

It should be taken into account, however, that the use of IHC screening to select cases for BRCA1 mutational analysis may have some limitations. In fact, although the majority of BRCA1-mutant tumours lack ER expression, several studies have shown that a small number retain ER positivity [26,27]. In addition, whereas the ER negative status is a highly sensitive predictor (ER-positive cases are rarely BRCA1 mutation carriers), this parameter has a limited specificity [1].

In this series of selected patients, a high prevalence of BRCA1 pathogenetic mutations was observed in FBCs with double or single negative receptorial status, showing that the evaluation of IHC characteristics can be useful not only for therapeutic purposes, but also for the identification of a subgroup of patients having a higher probability of being carriers of BRCA1 deleterious mutations. Furthermore, the evaluation of HER2 expression may also allow for a reduction of the number of patients who should undergo BRCA1 mutation screening.

## Conclusion

The IHC analysis by Allred's method, a rapid and easily performed test, can be used before performing expensive mutation screening in order to select the high-risk cases who are most likely to carry a deleterious BRCA1 mutation. In patients selected by family history, the analysis of the cancer IHC phenotype allows the prediction of the BRCA1 genotype with a very high sensitivity and specificity.

About 90% of BRCA1 related cancers are ER negative and/or PR negative with weak HER2 expression [4,5,19-21]. Although the IHC evaluation successfully allowed the limitation of the number of patients to be molecularly tested, a small proportion of cases carrying the BRCA1 mutations retained the ER expression.

Interestingly, our data confirm that the incidence of BRCA1 mutations in the ER and/or PR positive cancers is low. On these grounds, our combined selection approach could significantly reduce the number of patients who should undergo BRCA1 mutational analysis, with the possible limitation of missing one true BRCA1 mutation carrier every 100 patients selected by breast cancer familial history alone. Analysis of a larger series of cases is however required to validate the proposed selection approach.

## Competing interests

The authors declare that they have no competing interests.

## Authors' contributions

GM performed the genetic counselling and drafted the manuscript; AVi performed the molecular genetic studies and was involved in drafting and revising the paper; LDP performed the BRCA test; CDG, LN, SS, DLand AV collected the clinical data; VC, TPperformed the immunohistochemical characterization; RD performed the genetic counselling and was involved in drafting and revising the paper. All authors read and approved the final manuscript.

## Pre-publication history

The pre-publication history for this paper can be accessed here:

http://www.biomedcentral.com/1471-2407/9/360/prepub
